# Effect of Dietary Meat Content on Weight Gain, Mortality, and Pre-Pupal Rate in Black Soldier Fly (*Hermetia illucens*) Larvae

**DOI:** 10.3390/insects13030229

**Published:** 2022-02-25

**Authors:** Kiyonori Kawasaki, Mami Ohkawa, Junliang Zhao, Kiminobu Yano

**Affiliations:** 1 Department of Applied Biological Science, Faculty of Agriculture, Kagawa University, Ikenobe 2393, Miki-cho, Kita-gun, Kagawa 761-0795, Japan; td1364@pref.kagawa.lg.jp (M.O.); cho.shunryo@kagawa-u.ac.jp (J.Z.); 2 University Farm, Kagawa University, Showa 300-2, Sanuki, Kagawa 769-2304, Japan; yano.kiminobu@kagawa-u.ac.jp

**Keywords:** *Hermetia illucens*, waste meat processing, nitrogen-free extract

## Abstract

**Simple Summary:**

Recently, using insects to process food waste has attracted much attention from researchers. In this study, we investigated the extent to which meat can be included in the diets of immature black soldier flies. Based on our results, it was found that the meat content should be less than 80%. In addition, previous studies have shown that the protein and fat content of immature black soldier fly diets are important for growth and survival. However, it became clear in the present study that it is necessary to pay more attention to the nitrogen-free extract content of a diet than to the protein and fat content.

**Abstract:**

This study aimed to determine the protein content and the ratio of meat that can be added to the diet of black soldier fly (BSF) larvae for the sustainable recycling of food waste using insects. We conducted experiments feeding refined diets to BSF larvae with adjusted protein content and diets with minced pork, and analyzed the correlations between dietary nutrients and larval weight gain, mortality, and pre-pupal rate. The nutrient that was positively correlated with increased larval body weight and pre-pupal rate in both experiments was nitrogen-free extract (NFE). Diets with high concentrations of minced pork showed higher mortality of BSF larvae and were negatively correlated with dietary NFE content. It has been suggested that BSF larvae have reduced body weight and survival due to excessive protein and fat in their diet. Depending on the raw material and ratio of food waste, it may be necessary to adjust dietary protein and fat contents before treatment. The results of this study suggest that the NFE content of a larval diet influences the larval weight gain, mortality, and pre-pupal rate of BSF larvae in a great extent, a finding that has not been reported by previous studies.

## 1. Introduction

The use of insects as a novel food waste processing technology is being promoted worldwide [1,2,3]. Black soldier fly (BSF; *Hermetia illucens*) larvae eat a wide range of organic materials, such as food waste and livestock manure, and sixth instar larvae can be used as feed for pets, fish, poultry, and pigs [4,5,6,7,8,9,10].

Insects are attracting attention as feed ingredients for reasons other than their contribution to the resource recycling process [4]. Insects can be a substitute for fishmeal, which is used as an animal protein feed in aquaculture and livestock farming [11,12,13,14,15,16,17]. The price of fishmeal, a natural resource, fluctuates because its production is affected by climate change and other factors. Hence, insects that can be stably bred provide an alternative feed ingredient [17,18].

The BSF can be fed on organic wastes with high water and salt contents, and its larval growth period is short (less than 30 days), making it a promising candidate for production as an animal protein feed ingredient [16,19]. There are many companies globally that are involved in BSF production and use plant wastes, such as agro-processing residues, as a diet for BSF [4,20].

In European countries, laws and regulations stipulate that only plants and unprocessed milk and eggs can be used as a feeding material for insects, while fish, meat, slaughterhouse waste, catering waste, and livestock manure cannot be used [21]. These waste products may contain substances (drugs and pathogens) that are harmful to humans and insects and, therefore, should not be used in insect diets. Particularly, it is thought that processed animal proteins (PAPs) have the potential to cause transmissible spongiform encephalopathies (TSEs) and, thus, should not be used in feed for insects that could be used as animal feed ingredients. However, from the viewpoint of resource recycling, food waste such as fish, meat, and catering surplus can be used as a diet for insects, and research is being conducted to determine how to safely and efficiently produce insects fed on these food waste products.

A previous study reported that feeding raw meat or fish to BSF larvae increases their mortality due to excessive protein levels in their diet [22]. Therefore, it is necessary to combine other materials with high-protein food waste when producing feed for BSF larvae. Therefore, in this study, we aimed to determine the protein content and the ratio of waste meat that can be added to the diet of BSF larvae for the sustainable recycling of food waste using insects.

## 2. Materials and Methods

### 2.1. Experiment 1

#### 2.1.1. Larval Rearing Conditions

BSF eggs were collected from the farm at Kagawa University, Kagawa, Japan and hatched in a campus laboratory. BSF eggs that had been spawned in the plastic crevices of sorghum roll bale silos on the farm were placed in sterile 1.5 mL microtubes and transported to the laboratory. Larvae were hatched from the eggs in an incubator (MIR-154-PJ, PHC Holdings, Tokyo, Japan) set at 28 °C. After hatching, the BSF larvae were reared according to a previous study [23] on a basal diet (26.5% wheat germ, 15.9% commercial rabbit feed (LabDiet 5322, PMI Nutrition International, St. Louis, MO, USA), 2.6% dried yeast, and 55.0% deionized water) at a dose of 1 g/head, and were used for testing once they reached the age of 7 d old.

Fifty larvae per container were placed in 32 plastic containers (9 cm in diameter × 8 cm in height, 360 mL in volume) with ventilation openings and a piece of nylon mesh (70 mesh) under the lid. Larvae were allocated into eight groups (one for each experimental diet), and each group was replicated four times. The containers were kept in an incubator (MIR-154-PJ, PHC Holdings, Tokyo, Japan) for 14 d. The rearing temperature was 28 °C, and the light/dark periods were 12 h each.

#### 2.1.2. Diets

The experimental diets contained different crude protein percentages (CP), and a basal diet was used as the control (C). The CP of the raw diets was based on that of pork loin (approximately 22% CP) [24] and was formulated in gradual increments of 2%, from CP10 (10% CP as fed basis) to CP22 (22% CP as fed basis). The compositions of the experimental diets are shown in Table 1.

Experimental diets were passed through a sieve (2 mm) and then mixed with a spoon for approximately 5 min. The gross energy (GE) of the diet was adjusted to closely align with the CP value of each group.

#### 2.1.3. Body Weight Gain, Mortality, and Pre-Pupal Rate

Larvae were weighed before the experiment (7 days old) and after rearing for 14 days (21 days old). The larvae were weighed after being wiped with a paper towel to remove dirt and water.

Larval mortality was calculated by counting the number of living larvae at the end of the experiment.

At the end of the experiment, the appearance of the larvae was evaluated, and the blackened larvae were considered to be prepupa. The prepupa were counted and the percentage of prepupa in the living BSF larvae was calculated.

#### 2.1.4. Processing of Larvae

After completion of the rearing test, all the BSF larvae were collected from each container. The collected larvae were washed with deionized water, separated from dirt and water with a paper towel, and then killed by freezing at −20 °C. Before the analysis of larval proximate composition, the larvae were cut in half and dried with a freeze dryer (FDU-2200, EYELA, Tokyo, Japan). Freeze-dried larvae were ground in a laboratory blender (7011HS, Waring Laboratory Science, Stamford, CT, USA) and used for further analysis.

#### 2.1.5. Analysis of Proximate Composition

The proximate composition of the diet was analyzed according to the AOAC method [25]. Briefly, the following AOAC methods were used to analyze the proximate feed composition: moisture content (AOAC 930.15); ash content (AOAC 942.05); crude protein content (AOAC 984.13); crude fat content (AOAC 920.39C); and crude fiber content (AOAC 962.09). The larval proximate composition was analyzed using the same AOAC methods for crude protein and crude fat contents.

#### 2.1.6. Statistical Processing

All data obtained in this study were statistically processed using IBM SPSS Statistics version 26.0 [26]. The Kruskal–Wallis test was performed, and if a significant difference was found, the Dunn–Bonferroni post hoc method was performed to compare differences among the groups. Correlations between the body weight gain, mortality, and pre-pupal rate of BSF larvae and dietary nutrient composition were investigated using Spearman’s rank correlation coefficient.

### 2.2. Experiment 2

In Experiment 2, minced pork was added to the diet, and larval rearing tests were conducted. The ratios of minced pork in the diets are shown in Table 2. The minced pork was purchased from a supermarket on the day of experimental diet preparation. Sixty larvae per container were placed in 32 plastic containers. The determination of other parameters including larval processing, larval performance, and composition analysis were carried out as described in Experiment 1.

## 3. Results

### 3.1. Experiment 1

#### 3.1.1. Growth Results

The body weight gain, mortality, and pre-pupal rate of BSF larvae during Experiment 1 are shown in Table 3. Body weight gain was significantly higher in the CP10 group than in the CP22 group (*p* < 0.05). However, no significant difference in mortality was found among the groups. In addition, the pre-pupal rates of the C and CP10 groups were significantly higher than those of the CP22 group (*p* < 0.05).

The correlations between the nutrient composition of the diets and the body weight gain, mortality, and pre-pupal rate of BSF larvae are shown in Table 4. The body weight gain of BSF larvae showed a strong positive correlation with the crude fat (EE) and nitrogen-free extract (NFE) contents of the diets (*p* < 0.001), and a strong negative correlation with the crude protein content (*p* < 0.001) of the diets. The pre-pupal rate showed a strong negative correlation with the crude protein content (*p* < 0.001), and a positive correlation with NFE content (*p* < 0.01) of the diets. No correlation was observed between mortality and the dietary nutrient composition.

#### 3.1.2. Larval Proximate Composition

The percentages of crude protein and ether extract of larvae analyzed at the end of Experiment 1 are shown in Table 5. No significant differences were found among groups.

### 3.2. Experiment 2

#### 3.2.1. Growth Results

The body weight gain, mortality, and pre-pupal rate of BSF larvae during Experiment 2 are shown in Table 6. Rations with different proportions of minced pork addition affected the larval body weight gain, mortality, and pre-pupal rate. The M100 group showed lower body weight gain than the other groups and the difference was significant compared to the M70 and M80 groups (*p* < 0.05). The M80, M90, and M100 groups showed high mortality, and there was a significant difference between the mortality of the M60 and M100 groups (*p* < 0.05). Moreover, no pre-pupal larvae appeared in the M90 and M100 groups, and their pre-pupal rates were significantly lower than those of the M50 and M60 groups (*p* < 0.05).

The correlations between the nutrient composition of the diets and the body weight gain, mortality, and pre-pupal rate of BSF larvae in Experiment 2 are shown in Table 7. Body weight gain was significantly related to mortality (*p* < 0.05), but not to the pre-pupal rate and dietary nutrient composition. The mortality rate was positively correlated with the crude protein (*p* < 0.01) and crude fat contents (*p* < 0.05) of the diet, whereas it was negatively correlated with the crude fiber (*p* < 0.01), crude ash, and NFE contents (*p* < 0.05) of the diet. The pre-pupal rate showed a strong positive correlation with the crude fiber, crude ash, and NFE content of the diet (*p* < 0.001), and a strong negative correlation with the crude protein and crude fat contents of the diet (*p* < 0.001).

#### 3.2.2. Larval Proximate Composition

The percentages of crude protein and ether extract of the larvae were analyzed at the end of the experiment 2, and the results are shown in Table 8. The crude protein content was similar among the groups, and the M90 and M100 groups showed the greater numerical values. However, there was no significant difference in either the crude protein or fat content among groups.

## 4. Discussion

In the present study, we reared BSF larvae on diets containing different protein contents, and examined the effects on larval growth performance, to clarify the extent to which meat can be included in their diet. In addition, we reared BSF larvae on several diets with different pork contents and examined their growth performance under similar experimental conditions.

The results of Experiment 1 showed that the protein and NFE contents of the diets affected the body weight gain of BSF larvae. The results suggest that the protein content in the diet should constitute less than 50% of dry matter because the CP22 group showed the lowest body weight gain. Moreover, NFE content was positively correlated with both body weight gain and pre-pupal rate, suggesting that BSF larvae require saccharides for their growth if NFE is regarded as a carbohydrate. The different effects of dietary protein and NFE content on body weight gain in the two experiments may be explained by fat content differences among the diets. The result that body weight of BSF larvae was decreased by feeding only minced pork is in agreement with the results of Hirayasu et al. [22].

Since the mortality rate was higher when adding 80% minced pork or more to the diet, the meat content included in the diet should be less than 80%. In addition, no significant difference in mortality was found among the test groups of Experiment 1 when increasing the protein content, whereas mortality was significantly affected in Experiment 2 when the minced pork was used, which was probably due to the significantly lower NFE content of the diets containing higher proportions of minced pork. The NFE content was less than 23% for the M80–M100 groups, where the mortality rate was higher than that of the other groups. In contrast, the diets in Experiment 1, which did not show any significant difference in mortality, contained more than 40% NFE in all groups. This suggests that the NFE content of the diet for BSF larvae should be at least 30%. It was reported that a protein:lipid:digestible carbohydrate ratio of 2:1:2 is advantageous for larval development [27], but in the present study, the influence of NFE was greater than that of protein and lipid. A previous study suggested that the survival rate of BSF was decreased when the crude protein content of the BSF diet was approximately 10% on a dry matter basis [28]. However, in the present study, the lowest crude protein content in the BSF diet was 18.1% on a dry matter basis, which suggests that the BSF diet used in this study was not too low in protein content.

The pre-pupal rate was negatively correlated with the crude protein content and positively correlated with the NFE content of the experimental diets. Hence, if a high pre-pupal rate indicates a fast larval growth rate, it is important to consider crude protein and NFE contents when preparing a BSF larval diet. In the present study, the CP10 group showed the highest pre-pupal rate in Experiment 1, followed by the M50 and M60 groups in Experiment 2. The CP10 group showed the highest dietary NFE content in Experiment 1, whereas the highest dietary NFE content in Experiment 2 was in the WB group, followed by the C group. Therefore, the NFE content in diets is important for ensuring a high pre-pupal rate, but the protein content should be more than 20%.

A previous study reported that the proximal composition of BSF larvae was altered by different raw materials of the feeding material [29]. However, in the present study, only the ratio of the raw materials was changed and, thus, the larval proximate composition showed no significant difference in crude protein content for any of the treatments, suggesting that the protein content of BSF larvae is independent of the protein content in their diet. This result is in agreement with the results of Danieli et al. [30]. Furthermore, the crude fat content of larvae was not significantly different among the groups, but the crude fat content of larvae increased in Experiment 2, suggesting that the crude fat content of larvae was affected by the feed ingredients to some extent. A previous study reported that high non-structural carbohydrate content in the larval diet did not result in high ether extract (EE) content in BSF larvae [31], and similar results were obtained in the present study with no correlation between NFE and EE.

## 5. Conclusions

Previous studies suggest that BSF larvae have reduced body weight and survival due to excessive protein and fat in their diet. Depending on the raw material and ratio of food waste included in the feed, it may be necessary to adjust the protein and fat contents before treatment. The results of this study suggest that the NFE content of a larval diet influences the larval weight gain, mortality, and pre-pupal rate of BSF larvae, which has not been reported by previous studies. In this study, minced pork was mixed into the diet for BSF larvae, and due to the fact that meat nutritional composition varies depending on the type of animal, it is necessary to investigate the effects on BSF growth, survival, and proximate composition when meat from a variety of animals is used in the diet for BSF.

## Figures and Tables

**Table 1:** Ingredients and proximate composition of diets in Experiment 1.

Ingredient	C	CP10	CP12	CP14	CP16	CP18	CP20	CP22
Wheat germ	26.5	0.0	0.0	0.0	0.0	0.0	0.0	0.0
Rabbit feed ^1^	15.9	0.0	0.0	0.0	0.0	0.0	0.0	0.0
Dried yeast	2.6	0.0	0.0	0.0	0.0	0.0	0.0	0.0
Wheat bran	0.0	8.2	9.7	12.2	12.8	13.7	16.1	17.9
Potato starch	0.0	14.4	12.2	9.9	8.6	7.5	5.2	3.0
Casein	0.0	8.6	11.3	13.9	15.3	16.6	19.2	21.9
Corn	0.0	13.8	11.8	9.0	8.3	7.2	4.5	2.2
Deionized water	55.0	55.0	55.0	55.0	55.0	55.0	55.0	55.0
Proximate composition (% of dry matter)								
CP	37.2	25.9	29.5	34.1	41.8	48.5	50.9	51.2
EE	1.9	2.7	2.4	2.1	2.4	2.1	1.9	1.9
CF	5.9	1.5	1.3	1.2	1.4	1.8	2.1	2.6
CA	6.6	1.6	1.8	2.2	2.2	2.2	2.6	2.8
NFE	48.4	68.3	65.0	60.3	52.2	45.3	42.5	41.5
GE (Mcal/kg) ^2^	4.6	4.7	4.7	4.8	4.9	5.0	5.0	5.0

C: control; CP: crude protein; EE: ether extract; CF: crude fiber; CA: crude ash; NFE: nitrogen-free extract; GE: gross energy. ^1^ LabDiet 5322 (PMI Nutrition International), ^2^ GE = (CP × 5.67 + EE × 9.68 + NFE × 4.25 + CF × 4.90)/100.

**Table 2:** Ingredients and proximate composition of diets in Experiment 2.

Ingredient	C	WB	M50	M60	M70	M80	M90	M100
Wheat germ	22.4	0.0	0.0	0.0	0.0	0.0	0.0	0.0
Rabbit feed ^1^	13.4	0.0	0.0	0.0	0.0	0.0	0.0	0.0
Dried yeast	2.2	0.0	0.0	0.0	0.0	0.0	0.0	0.0
Wheat bran	0.0	39.9	28.5	24.9	20.7	15.4	8.7	0.0
Minced pork	0.0	0.0	28.5	37.4	48.2	61.5	78.3	100.0
Deionized water	62.0	60.1	43.0	37.7	31.1	23.1	13.0	0.0
Composition (% of dry matter)								
CP	37.2	18.1	28.3	31.4	35.3	40.0	46.0	53.8
EE	1.9	5.0	16.0	19.4	23.6	28.7	35.2	43.6
CF	5.9	10.9	7.8	6.8	5.7	4.2	2.4	0.0
CA	6.6	5.9	4.9	4.6	4.3	3.9	3.3	2.6
NFE	48.4	60.1	43.0	37.6	31.2	23.2	13.1	0.0
GE (Mcal/kg) ^2^	4.6	4.6	5.4	5.6	5.9	6.2	6.7	7.3

C: control; WB: wheat bran; M: minced pork; CP: crude protein; EE: ether extract; CF: crude fiber; CA: crude ash; NFE: nitrogen-free extract; GE: gross energy. ^1^ LabDiet 5322 (PMI Nutrition International), ^2^ GE = (CP × 5.67 + EE × 9.68 + NFE × 4.25 + CF × 4.90)/100.

**Table 3:** Body weight gain, mortality, and pre-pupal rate of BSF larvae in Experiment 1.

Group	Body Weight Gain (g)	Mortality (%)	Pre-Pupal Rate (%)
C	1.178 ± 0.078 ^a,b^	0.5 ± 0.5	67.5 ± 5.8 ^a^
CP10	1.899 ± 0.065 ^a^	6.5 ± 2.2	71.0 ± 2.3 ^a^
CP12	1.677 ± 0.17 ^a,b^	7.0 ± 2.1	49.5 ± 3.7 ^a,b^
CP14	1.348 ± 0.020 ^a,b^	4.5 ± 2.6	31.8 ± 2.2 ^a,b^
CP16	1.257 ± 0.047 ^a,b^	4.0 ± 2.3	19.3 ± 8.5 ^a,b^
CP18	1.265 ± 0.044 ^a,b^	2.7 ± 1.3	16.0 ± 8.7 ^a,b^
CP20	1.157 ± 0.039 ^a,b^	5.0 ± 3.1	10.8 ± 3.7 ^a,b^
CP22	1.003 ± 0.077 ^b^	5.5 ± 0.5	3.3 ± 2.1 ^b^

C: control; CP: crude protein. Data: Mean ± SE; *n* = 4; different letters indicate significant differences; *p* < 0.05.

**Table 4:** Spearman’s correlations in Experiment 1.

Variable	BWG	Mortality	Pre-Pupal Rate	CP	EE	CF	CA	NFE
BWG	-	-	-	-	-	-	-	-
Mortality	0.284	-	-	-	-	-	-	-
Pre-pupal rate	0.544 **	−0.212	-	-	-	-	-	-
CP	−0.821 ***	−0.022	−0.791 ***	-	-	-	-	-
EE	0.805 ***	0.294	0.447 *	−0.805 ***	-	-	-	-
CF	−0.624 ***	−0.249	−0.097	0.571 ***	−0.756 ***	-	-	-
CA	−0.799 ***	−0.316	−0.348	0.762 ***	−0.952 ***	0.833 ***	-	-
NFE	0.839 ***	0.103	0.699 ***	−0.976 ***	0.903 ***	−0.69 ***	−0.857 ***	-

BWG: body weight gain; CP: crude protein; EE: ether extract; CF: crude fiber; CA: crude ash; NFE: nitrogen-free extract. * *p* < 0.05, ** *p* < 0.01, *** *p* < 0.001.

**Table 5:** Crude protein and ether extract of BSF larvae in Experiment 1.

Group	CP (%)	EE (%)
C	66.18 ± 2.10	16.77 ± 3.13
CP10	64.09 ± 2.89	24.31 ± 2.77
CP12	65.42 ± 2.65	19.15 ± 3.48
CP14	65.86 ± 2.00	22.28 ± 2.00
CP16	64.91 ± 2.57	17.72 ± 1.42
CP18	66.77 ± 1.71	21.45 ± 1.95
CP20	65.73 ± 0.65	19.68 ± 1.82
CP22	67.50 ± 0.92	15.40 ± 3.27

C: control; CP: crude protein; EE: ether extract. Data: Mean ± SE; *n* = 4.

**Table 6:** Body weight gain, mortality, and pre-pupal rate of BSF larvae in Experiment 2.

Group	Body Weight Gain (g)	Mortality (%)	Pre-Pupal Rate (%)
C	1.946 ± 0.117 ^a,b^	4.5 ± 1.0 ^a,b^	32.5 ± 2.9 ^a,b^
WB	1.860 ± 0.082 ^a,b^	2.3 ± 0.3 ^a,b^	46.8 ± 3.6 ^a,b^
M50	1.953 ± 0.048 ^a,b^	3.3 ± 0.6 ^a,b^	54.5 ± 1.7 ^a^
M60	2.142 ± 0.047 ^a,b^	1.0 ± 0.6 ^a^	50.8 ± 1.3 ^a^
M70	2.400 ± 0.082 ^a^	6.8 ± 4.5 ^a,b^	23.0 ± 7.5 ^a,b^
M80	2.275 ± 0.069 ^a^	12.3 ± 6.5 ^a,b^	5.3 ± 3.8 ^a,b^
M90	1.822 ± 0.061 ^a,b^	11.8 ± 8.5 ^a,b^	0.0 ± 0.0 ^b^
M100	1.001 ± 0.133 ^b^	44.3 ± 7.0 ^b^	0.0 ± 0.0 ^b^

C: control; WB: wheat bran; M: minced pork. Data: Mean ± SE; *n* = 4; different letters indicate significant differences; *p* < 0.05.

**Table 7:** Spearman’s correlations in Experiment 2.

Variable	BWG	Mortality	Prepupa Rate	CP	EE	CF	CA	NFE
BWG	-	-	-	-	-	-	-	-
Mortality	−0.395 *	-	-	-	-	-	-	-
Prepupa rate	0.195	−0.557 ***	-	-	-	-	-	-
CP	−0.272	0.535 **	−0.896 ***	-	-	-	-	-
EE	−0.152	0.359 *	−0.741 ***	0.762 ***	-	-	-	-
CF	0.186	−0.507 **	0.907 ***	−0.976 ***	−0.857 ***	-	-	-
CA	0.152	−0.359 *	0.741 ***	−0.762 ***	−1.000 ***	0.857 ***	-	-
NFE	0.139	−0.407 *	0.784 ***	−0.857 ***	−0.976 ***	0.929 ***	0.976 ***	-

BWG: body weight gain; CP: crude protein; EE: ether extract; CF: crude fiber; CA: crude ash; NFE: nitrogen-free extract. * *p* < 0.05, ** *p* < 0.01, *** *p* < 0.001.

**Table 8:** Crude protein and ether extract of BSF larvae in Experiment 2.

Group	CP (%)	EE (%)
C	61.80 ± 2.31	25.69 ± 2.89
WB	59.17 ± 2.13	28.81 ± 4.06
M50	62.07 ± 2.14	32.07 ± 3.35
M60	62.16 ± 1.77	29.28 ± 2.44
M70	61.20 ± 2.28	30.77 ± 5.16
M80	56.95 ± 1.52	34.08 ± 4.35
M90	57.76 ± 3.41	43.84 ± 3.92
M100	59.62 ± 3.17	42.11 ± 4.63

C: control; WB: wheat bran; M: minced pork; CP: crude protein; EE: ether extract. Data: Mean ± SE; *n* = 4.

## Data Availability

Data are contained within the article.
